# Supplementary material for: Splice-Junction-Based Mapping of Alternative Isoforms in the Human Proteome
Source: Cell Rep. Author manuscript; Available in PMC 2020 Jan 15. (PMC6961840; doi:10.1016/j.celrep.2019.11.026)
Supplement: 2 [file NIHMS1546469-supplement-2.zip › DF1/PXD010154/Lung/Lung_44_TAF1C_KPRMGLTCPGSWPSAAAREP.pdf]

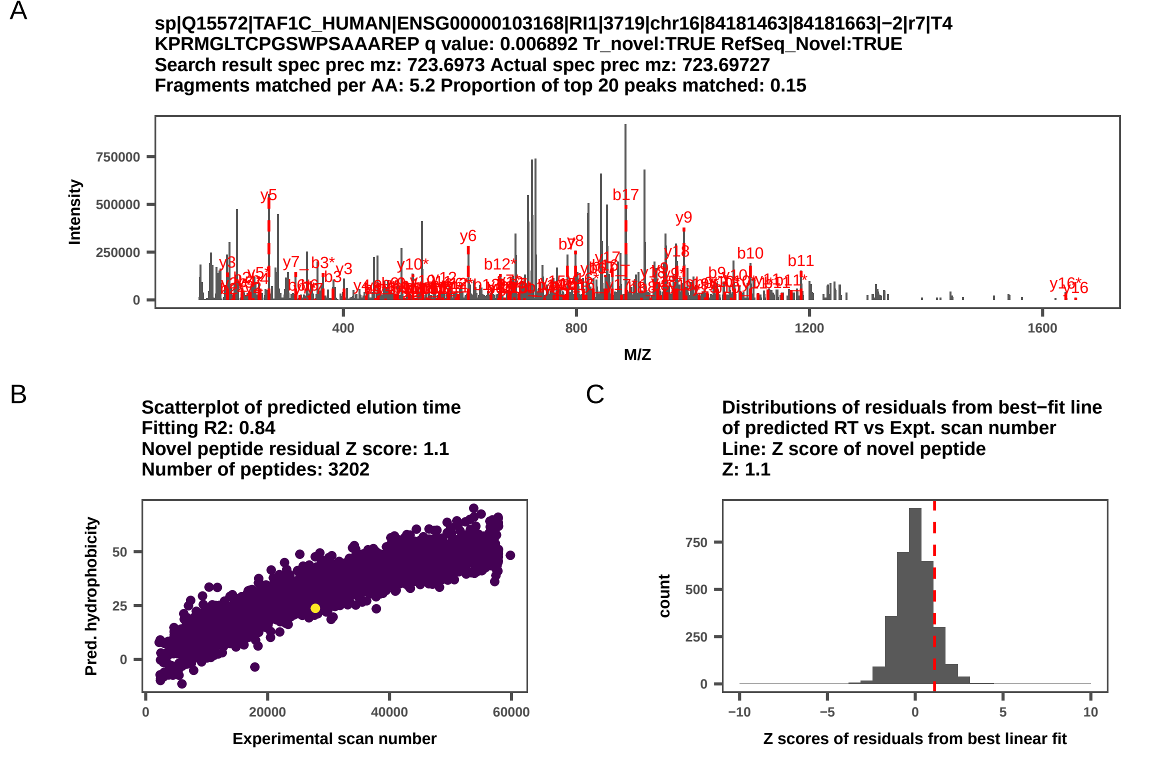
A
sp|Q15572|TAF1C_HUMAN|ENSG00000103168|RI1|3719|chr16|84181463|84181663|−2|r7|T4
KPRMGLTCPGSWPSAAAREP q value: 0.006892 Tr_novel:TRUE RefSeq_Novel:TRUE
Search result spec prec mz: 723.6973 Actual spec prec mz: 723.69727
Fragments matched per AA: 5.2 Proportion of top 20 peaks matched: 0.15
Intensity
750000
500000
250000
0
400
800
1200
1600
M/Z
y5
b17
y9
y6
y10*
b12*
y7
b3*
y3
y18
b10
b11
y16*
y16
B
Scatterplot of predicted elution time
Fitting R2: 0.84
Novel peptide residual Z score: 1.1
Number of peptides: 3202
Pred. hydrophobicity
50
25
0
0
20000
40000
60000
Experimental scan number
C
Distributions of residuals from best−fit line
of predicted RT vs Expt. scan number
Line: Z score of novel peptide
Z: 1.1
count
750
500
250
0
−10
−5
0
5
10
Z scores of residuals from best linear fit
